# Supplementary material for: Promotech: a general tool for bacterial promoter recognition
Source: Genome Biol. 2021 Nov 17;22:318. doi: 10.1186/s13059-021-02514-9 (PMC8597233; doi:10.1186/s13059-021-02514-9)
Supplement: Supplementary file 1 — Additional file 1 The Additional file 1 is a PDF file that includes the following tables: Table S1 — A summary of promoter prediction approaches in the last twelve years. Table S2 — The number of TSS per each bacterium in the training data set. Table S3 — The number of TSS per each bacterium in the validation data set. Table S4 — RNN hyper-parameters. Supplementary Figs. 1 - 4 — PRC and ROC per model and per bacterium when requiring 10% sequence overlap between predicted promoters and actual promoters to count them as true positives. [file 13059_2021_2514_MOESM1_ESM.pdf]

Promotech's additional file

Ruben Chevez-Guardado and Lourdes Peña Castillo
Memorial University of Newfoundland

September 28, 2021

Contents

1 **Computational approaches for bacterial promoter recognition** 2

2 **Number of instances per bacterium in training and validation data sets** 4

3 **Whole-genome promoter search requiring 10% sequence overlap between predicted promoters and actual promoters** 6

4 **RNN's hyper-parameters** 8

Bibliography 9

1 Computational approaches for bacterial promoter recognition

Table 1: A number of promoter prediction approaches published within the last twelve years. Columns are methods' name and publication year, brief description of approach used to detect promoters, and target organism(s).

Name	Approach	Target Organism(s)
Selector [1] (2021)	Stacked-ensemble approach	*E. coli*
iPromoter-BnCNN [2] (2020)	CNN-based classifier	*E. coli*
MULTiPLy [3] (2019)	SVM	*E. coli*
iProEP [4] (2019)	Support vector machine (SVM)	*H. sapiens*<br>*D. melanogaster*<br>*C. elegans*<br>*B. subtilis*<br>*E. coli*
iPro70-FMWin [5] (2018)	Sequence-based features extracted for "multiple windows" and logistic regression	*E. coli*
IBPP [6] (2018)	SVM	*E. coli*
iPromoter-2L [7] (2018)	Multi-window-based pseudo K-tuple nucleotide composition	*E. coli*
70ProPred [8] (2018)	SVM	*E. coli*
G4PromFinder [9] (2018)	Detection of AT-rich element and G-quadruplex motifs	*S. coelicolor A3(2)*<br>*P. aeruginosa PA14*

bTSSfinder [10] (2017)	Position weight matrices (PWM) and neural networks	*E. coli K12*<br>*Nostoc sp. PCC 7120*<br>*Synechocystis sp. PCC 6803*<br>*S. elongatus PCC 6301*
CNNProm [11] (2017)	Convolutional Neural Networks (CNN)	*E. coli*<br>*B. subtilis*<br>*Homo sapiens*<br>*Mus musculus*<br>*Arabidopsis*
vw Z-curve [12] (2012)	Variable-window Z-curve for frequencies of k-nucleotides and partial least squares classifier	*E. coli*<br>*B. subtilis*
PePPER [13] (2012)	MEME motif search for the Pribnow box DNA pattern	*L. lactis*<br>*E. coli*<br>*B. subtilis*
BacPP [14] (2011)	Neural networks	*E. coli*
PromPredict [15] (2010)	Thresholds on sequences' average free energy	*E. coli*<br>*B. subtilis*<br>*M. tuberculosis*
BPROM [16] (2010)	Linear Discriminant Analysis (LDA)	*E. coli*

2 Number of instances per bacterium in training and validation data sets

Table 2: Training data sets. Data sets are given in the same order as in Table 10 in the manuscript

BACTERIA ID	PROMOTER SEQUENCES	NON-PROMOTER SEQUENCES
ECOLI	248	2,773
ECOLI_2	2,636	26,147
HPYLORI	1,877	18,273
HPYLORI_2	448	4,448
CJEJUNI	269	2,674
CJEJUNI_2	1,881	18,298
CJEJUNI_3	2,140	20,736
CJEJUNI_4	1,919	18,619
CJEJUNI_5	1,973	19,173
SPYOGENE	891	8,754
STYPHIRMURIUM	1,869	18,464
CPNEUMONIAE	530	5,221
SONEIDENSIS	4,728	46,511
LINTERROGANS	2,791	27,979
SCOELICOLOR	3,566	35,170
**SUBTOTAL**	27,766	273,240
**TOTAL**	301,006	

Table 3: Balanced test data sets. These data sets were not used during training. They were used to assess Promotech's performance on independent data and compare its performance with that of existing tools.

BACTERIA ID	PROMOTER SEQUENCES	NON-PROMOTER SEQUENCES
MYCOBACTER	4,054	3,978
CLOSTRIDIUM	1,187	1,177
RHODOBACTER	5,374	5,207
BACILLUS	1,064	1,055
**SUBTOTAL**	11,615	11,417
**TOTAL**	23,032	

3 Whole-genome promoter search requiring 10% sequence overlap between predicted promoters and actual promoters

Figure 1: A comparison between the AUPRC and AUROC requiring 10% sequence overlap between predicted promoters and actual promoters in the *Mycolicibacterium smegmatis* str. MC2 155 bacterium. Column **(a)** shows the PR curves per model and **(b)** shows the ROC curves.

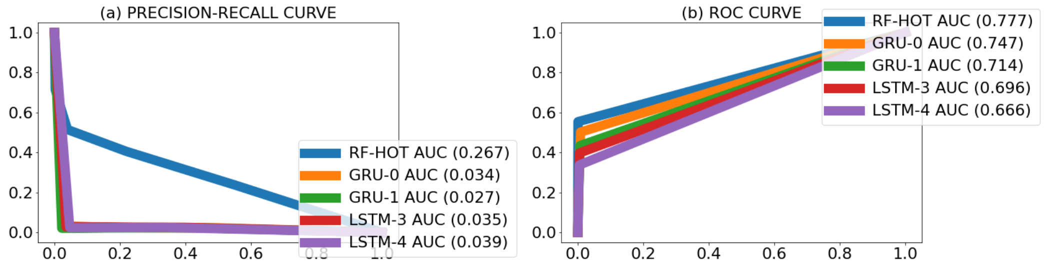

Figure 2: A comparison between the AUPRC and AUROC requiring 10% sequence overlap between predicted promoters and actual promoters in the *Lachnoclostridium phytofermentans* ISDg bacterium. Column **(a)** shows the PR curves per model and **(b)** shows the ROC curves.

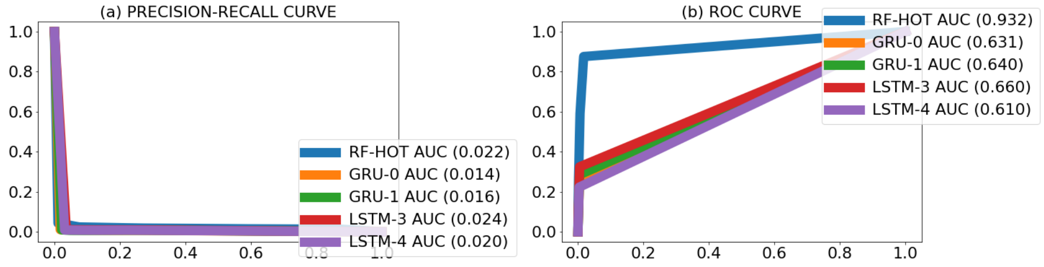

Figure 3: A comparison between the AUPRC and AUROC requiring 10% sequence overlap between predicted promoters and actual promoters in the *Rhodobacter capsulatus* SB 1003 bacterium. Column **(a)** shows the PR curves per model and **(b)** shows the ROC curves.

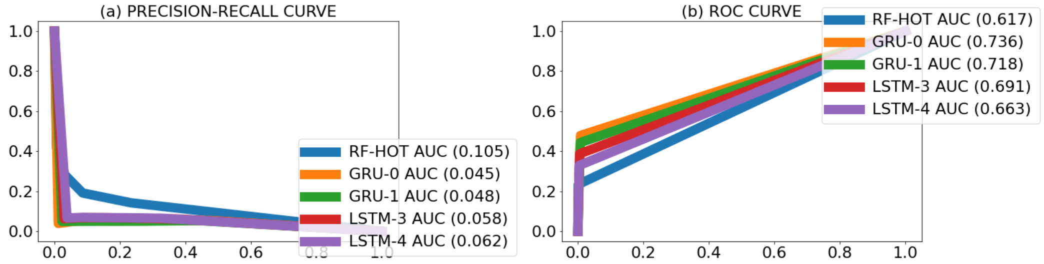

Figure 4: A comparison between the AUPRC and AUROC requiring 10% sequence overlap between predicted promoters and actual promoters in the *Bacillus amyloliquefaciens* XH7 bacterium. Column **(a)** shows the PR curves per model and **(b)** shows the ROC curves.

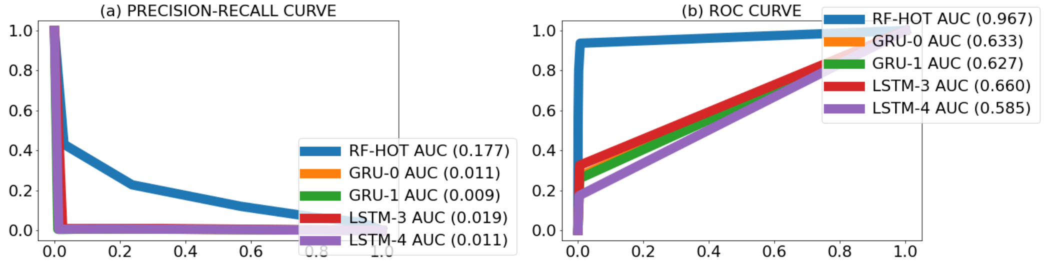

4 RNN's hyper-parameters

Table 4: Hyper-parameter setting used in the RNN's architecture and training.

**Hyper-parameter**	**Value**
Number of embedding units	50
Number of recurrent units	100
Type of recurrent units	LSTM or GRU
Number of fully-connected units	100
Number of hidden dense layers	0, 1, 2, 3, or 4
Dropout rate	0.2
Epochs	50
Batch size	10
Activation function	Sigmoid
Optimizer	Adam
Loss function	Binary cross-entropy

References

[1] Fuyi Li, Jinxiang Chen, Zongyuan Ge, Ya Wen, Yanwei Yue, Morihiro Hayashida, Abdelkader Baggag, Halima Bensmail, and Jiangning Song. Computational prediction and interpretation of both general and specific types of promoters in *Escherichia coli* by exploiting a stacked ensemble-learning framework. *Brief Bioinform*, 22(2):2126–2140, Mar 2021.

[2] Ruhul Amin, Chowdhury Rafeed Rahman, Sajid Ahmed, Md Habibur Rahman Sifat, Md Nazmul Khan Liton, Md Moshiur Rahman, Md Zahid Hossain Khan, and Swakkhar Shatabda. iPromoter-BnCNN: a novel branched CNN-based predictor for identifying and classifying sigma promoters. *Bioinformatics*, 36(19):4869–4875, 12 2020.

[3] Meng Zhang, Fuyi Li, Tatiana T Marquez-Lago, André Leier, Cunshuo Fan, Chee Keong Kwoh, Kuo-Chen Chou, Jiangning Song, and Cangzhi Jia. MULTiPly: a novel multi-layer predictor for discovering general and specific types of promoters. *Bioinformatics*, 35(17):2957–2965, 2019.

[4] Hong-Yan Lai, Zhao-Yue Zhang, Zhen-Dong Su, Wei Su, Hui Ding, Wei Chen, and Hao Lin. iProEP: a computational predictor for predicting promoter. *Molecular Therapy-Nucleic Acids*, 17:337–346, 2019.

[5] Md Siddiqur Rahman, Usma Aktar, Md Rafsan Jani, and Swakkhar Shatabda. ipro70-fmwin: identifying sigma70 promoters using multiple windowing and minimal features. *Molecular Genetics and Genomics*, pages 1–16, 2018.

[6] Sheng Wang, Xuesong Cheng, Yajun Li, Min Wu, and Yuhua Zhao. Image-based promoter prediction: a promoter prediction method based on evolutionarily generated patterns. *Scientific reports*, 8(1):1–9, 2018.

[7] Bin Liu, Fan Yang, De-Shuang Huang, and Kuo-Chen Chou. iPromoter-2L: a two-layer predictor for identifying promoters and their types by multi-window-based PseKNC. *Bioinformatics*, 34(1):33–40, 01 2018.

[8] Wenying He, Cangzhi Jia, Yucong Duan, and Quan Zou. 70propred: a predictor for discovering sigma70 promoters based on combining multiple features. *BMC Systems Biology*, 12(4):44, 2018.

[9] Marco Di Salvo, Eva Pinatel, Adelfia Talà, Marco Fondi, Clelia Peano, and Pietro Alifano. G4PromFinder: an algorithm for predicting transcription promoters in GC-rich bacterial genomes based on AT-rich elements and G-quadruplex motifs. *BMC bioinformatics*, 19(1):36, 2018.

[10] Ilham Ayub Shahmuradov, Rozaimi Mohamad Razali, Salim Bougouffa, Aleksandar Radovanovic, and Vladimir B Bajic. bTSSfinder: a novel tool for the prediction of promoters in *Cyanobacteria* and *Escherichia coli*. *Bioinformatics*, 33(3):334–340, 2017.

[11] Ramzan Kh Umarov and Victor V Solovyev. Recognition of prokaryotic and eukaryotic promoters using convolutional deep learning neural networks. *PloS one*, 12(2), 2017.

[12] Kai Song. Recognition of prokaryotic promoters based on a novel variable-window z-curve method. *Nucleic Acids Res*, 40(3):963–71, Feb 2012.

[13] Anne de Jong, Hilco Pietersma, Martijn Cordes, Oscar P Kuipers, and Jan Kok. PePPER: a webserver for prediction of prokaryote promoter elements and regulons. *BMC genomics*, 13(1):299, 2012.

[14] Scheila de Avila e Silva, Sergio Echeverrigaray, and Günther J L Gerhardt. BacPP: bacterial promoter prediction — a tool for accurate sigma-factor specific assignment in enterobacteria. *Journal of theoretical biology*, 287:92–99, 2011.

[15] Vetriselvi Rangannan and Manju Bansal. High-quality annotation of promoter regions for 913 bacterial genomes. *Bioinformatics*, 26(24):3043–3050, 2010.

[16] Victor Solovyevand Asaf Salamov and A Solovyevand. Automatic annotation of microbial genomes and metagenomic sequences. *Metagenomics and its applications in agriculture. Nova Science Publishers, Hauppauge*, pages 61–78, 2011.
