# Supplementary material for: Promotech: a general tool for bacterial promoter recognition
Source: Genome Biol. 2021 Nov 17;22:318. doi: 10.1186/s13059-021-02514-9 (PMC8597233; doi:10.1186/s13059-021-02514-9)
Supplement: Supplementary file 2 — Additional file 2 Review history. [file 13059_2021_2514_MOESM2_ESM.docx]

**Review history**

**First round of review**

**Reviewer 1**

In this work, the authors proposed a general bacterial promoter recognition method the model of which was trained on a large dataset of eight distinct bacterial species belonging to five different phyla. It is appreciable that the researchers have done much work on the data collection and the data set construction. However, there are some problems that have to be addressed in order to merit the publication in a high IF journal:
1) In the 'Collecting data' section, the authors assumed the promoter sequences recognized by Eσ is in the middle of this region. What is the basis for this assumption? There is no description of this part in the manuscript.
2) I can see very little that is new from the exploration of machine learning-based classification algorithms.
3) The word embedding representation of RNN is not clear. The manuscript only discussed the layers of RNN, but did not explain and explore other hyperparameters.
4) In addition, the authors make a great deal about feature analysis; however, they don't make use of the analysis results to improve the model performance.
5) I would recommend to provide an overall framework figure to make it easy for readers to understand the experimental procedures.

Overall, in my humble opinion, this work still needs further construction and improvement. As such, the current version should be rejected.

**Reviewer 2**

This study presented a general machine-learning-based method for bacterial promoter recognition. The approaches are appropriate to the aims of the study and the results are correct. I could see the value of their contribution, but they specifically highlight their models predict promoters in the proximity of actual promoters, it does not become clear to me how they can locate the position. In my humble opinion, the authors should give more explanation and experiments.
There are a number of points that need clarification as detailed below.
1)     How do they define 'tetra-nucleotide frequencies'?
2)     Why just chose RF and RNN as classifiers? Did you try other deep learning algorithms? Maybe it could then perform significantly (or at least) better than the current model?
3)     I know the rules you selected positive samples, and how about negative samples?
4)     Comprehensive comparison should be added, such as with model in PMID: 32363397.
5)     Apart from the sequence encoding features, can you consider any other features (that are not only sequence dependent) ?
6)     Regarding performance, did you look into AUC only? How is the performance with recall and precision?

**Authors’ response to reviewers**

We greatly appreciate the effort and insightful comments of the Reviewers. We have taken the comments from the Reviewers into consideration and feel our manuscript has been improved. Our detailed response to their comments is below.

Response to Reviewers

Reviewer #1: ===

In this work, the authors proposed a general bacterial promoter recognition method the model of which was trained on a large dataset of eight distinct bacterial species belonging to five different phyla. It is appreciable that the researchers have done much work on the data collection and the data set construction. However, there are some problems that have to be addressed in order to merit the publication in a high IF journal:

1) In the 'Collecting data' section, the authors assumed the promoter sequences recognized by Eσ is in the middle of this region. What is the basis for this assumption? There is no description of this part in the manuscript.

Eσ covers DNA roughly from -55 bp upstream to +15 bp relative to the TSS [Mejia-Almonte et al, Nature Reviews Genetics 2020]. It is known that promoter sequences are located before TSS thus the 15bp downstream of the TSS are not part of the promoter sequence. We needed then to remove these 15 bp downstream of the TSS, and for symmetry, we removed 15 bp from both sides. Additionally, Figs. 2 and 3 in [Mejia-Almonte et al, Nature Reviews Genetics 2020] show as the promoter region the 40bp upstream of the TSS. Note that as per Mejia-Almonte et al, we are considering as the promoter sequence Eσ binding site without the activator sites. We have clarified this in the manuscript with the following text: “Eσ covers DNA from roughly 55bp upstream to 15 bp downstream of the TSS [1]. As the promoter region is not located downstream of theTSS and Eσ covers 15bp downstream of the TSS, we excluded 15 bp from both sides of the region covered by Eσ, and took as the promoter sequence the 40bp upstream of the TSS.”

2) I can see very little that is new from the exploration of machine learning-based classification algorithms.

The goal of our study is to develop a species-independent machine-learning-based method for bacterial promoter identification shown to work on a wide range of bacterial species. To do this, we trained and evaluate our models on a large and diverse data set. This is in contrast with most existing bacterial promoter prediction methods which focus mostly on E. coli or on very few bacterial species. To achieve our goal, we explored two machine learning classification algorithms (random forests and recurrent neural networks) known to be suitable for sequence-derived features and optimized their most important hyper-parameters. It is out of the scope of this study to perform an exhaustive exploration of ML classification algorithms.

3) The word embedding representation of RNN is not clear. The manuscript only discussed the layers of RNN, but did not explain and explore other hyperparameters.

The word embedding is done in the first layer of the RNN, and the weights to obtain a numerical representation for each tetra-nucleotide are learnt during training (this is now illustrated in Figure 1 – Flowchart diagram). We performed a manual tuning of some hyperparameters (as described in Chapter 11 of the book “Deep Learning” by Ian Goodfellow, Yoshua Bengio and Aaron Courville) such as dropout rate. In Chapter 6 of the same book, the authors present empirical results showing the importance of depth on network performance, therefore we explored networks of different depths. To clarify the network architecture used, we are now including Table 5 in Additional File 1 with all the hyper-parameters of the RNN.

4) In addition, the authors make a great deal about feature analysis; however, they don't make use of the analysis results to improve the model performance.

We performed feature *importance* analysis to aid in the interpretation of the models. However, in the manuscript we used “Feature analysis” as the heading of this section. We apologize for the misunderstanding. Based on our analysis, all of the features are relevant for classification, and thus, there is no reason to remove any of them. The most relevant features are part of the Pribnow-Schaller box suggesting our random forests models learnt that presence of this motif is an important indicator of whether or not a sequence contains a promoter. We are now using the heading “Model interpretation” for this section, and have made clear the goal of this analysis by adding the text: “To interpret the models created, we performed feature importance analysis to find out motifs recognized by the models. To do this, we obtained the feature importance ranking from the RF models.”.

5) I would recommend to provide an overall framework figure to make it easy for readers to understand the experimental procedures.

Thanks for the suggestion! We have added Figure 1 in the manuscript illustrating our methodology.

Reviewer #2:

This study presented a general machine-learning-based method for bacterial promoter recognition. The approaches are appropriate to the aims of the study and the results are correct. I could see the value of their contribution, but they specifically highlight their models predict promoters in the proximity of actual promoters, it does not become clear to me how they can locate the position. In my humble opinion, the authors should give more explanation and experiments.

There are a number of points that need clarification as detailed below.

1) How do they define 'tetra-nucleotide frequencies'?

We have added the following definition of tetra-nucleotide in the manuscript: “To calculate the tetra-nucleotide frequencies vector of a given sequence, the number of occurrences of each possible 4-nucleotide DNA sequence (4-mer) in that sequence is divided by the total number of 4- mers in it.”.

2) Why just chose RF and RNN as classifiers? Did you try other deep learning algorithms? Maybe it could then perform significantly (or at least) better than the current model?

We selected random forests and recurrent neural networks because they have been shown to be suitable for sequence-derived features. Based on the literature, selecting one or few learning algorithms to evaluate as classifier(s) is standard practice in this and many other application areas. As we did not try all existing learning algorithms, we cannot discard the possibility that other learning algorithm might perform similarly to or better than our current model.

3) I know the rules you selected positive samples, and how about negative samples?

As explained in the section “Generating positive and negative instances sets”, negative instances are randomly selected genomic regions without overlap with the positive instances. This is also now illustrated in Figure 1.

4) Comprehensive comparison should be added, such as with model in PMID: 32363397.

We completely agree that the more approaches added to a comparative assessment, the better. Thus we tried to include several other recently published approaches to our benchmark.

However, several web sites and/or downloadable codes were not working properly during the time we undertook this revision. The approaches we tried to add are the following:

1)**SELECTOR (the approach suggested by the Reviewer). SELECTOR's web site has been down since we received the reviews (see image pasted below). On April 9th, we sent an email to the corresponding author but have not heard back from them.**
2) **iPromoter-2L (PMID:28968797). As the code for iPromoter-2L is not available on their website (**[**http://bioinformatics.hitsz.edu.cn/iPromoter-2L/),**](http://bioinformatics.hitsz.edu.cn/iPromoter-2L/)) **we submitted our sequences as Fasta file to iPromoter-2L website. iPromoter-2L sever returned an error (see image below).**

3)**iPromoter-BnCNN (PMID: 32614400). We could not access iPromoter-BnCNN's web site at the address provided (**[**http://103.109.52.8/iPromoter-BnCNN)**](http://103.109.52.8/iPromoter-BnCNN)) **in the corresponding manuscript (see image below).**

We also tried to obtain iPromoter-BnCNN's results submitting a text file with 81nt-long sequences in the format specified to iPromoter-BnCNN's code in Google Colab. Promoter-BnCNN's code crashed with an error (see image below).

4) iProEP (PMID: 31299595). We dowloaded their code and followed the instructions to run it in a linux computer. However, their code crashed with an error.
5) **CNNProm (PMID: 28158264). Their web site (**[**http://www.softberry.com/berry.phtml?**](http://www.softberry.com/berry.phtml) **topic=cnnpromoter_b&group=programs&subgroup=deeplearn) works perfectly; however, it does not take multi-sequence Fasta files. As our data sets consists of ~23 thousand sequences, submitting each of the them individually to CNNProm is infeasible.**

At the end, we were only able to add iPro70-FMWin (PMID: 30187132) to our comparative assessment. iPro70-FMWin was found by an independent benchmarking study (PMID: 32843538) to be the best performing promoter prediction tool, among eight tools assessed, in terms of accuracy and MCC for the identification of *E. coli* promoters.

We also evaluated the performance of Promotech on an *E. coli* data set used in several published manuscripts to evaluate promoter prediction tools.

5) Apart from the sequence encoding features, can you consider any other features (that are not only sequence dependent)?

We could use expression data to obtain features representing promoter activity; however, requiring any other data type for performing predictions would make Promotech not as widely applicable as we wish. Furthermore, most bacterial promoter prediction tools only require as input DNA sequences and we want to do the same.

6) Regarding performance, did you look into AUC only? How is the performance with recall and precision?

We look into area under the ROC curve (AUROC) and area under the precision-recall curve (AUPRC). Thus, the performance in terms of recall and precision can be seen from the precision-recall curves (PRCs) and AUPRC values included in the manuscript.

**Second round of review**

**Reviewer 1**

-

**Reviewer 2**

I am satisfied with the responses provided by the authors. So I recommend this paper to be publish in GB.
